# The role of urbanisation in the spread of *Aedes* mosquitoes and the diseases they transmit—A systematic review

**DOI:** 10.1371/journal.pntd.0009631

**Published:** 2021-09-09

**Authors:** Antonios Kolimenakis, Sabine Heinz, Michael Lowery Wilson, Volker Winkler, Laith Yakob, Antonios Michaelakis, Dimitrios Papachristos, Clive Richardson, Olaf Horstick

**Affiliations:** 1 Benaki Phytopathological Institute, Scientific Directorate of Entomology and Agricultural Zoology, Kifissia, Greece; 2 Heidelberg Institute of Global Health, Research to Practice Group, Heidelberg University, Heidelberg, Germany; 3 London School of Hygiene and Tropical Medicine, London, United Kingdom; 4 Department of Economic and Regional Development, Panteion University of Social and Political Sciences, Athens, Greece; Faculty of Science, Mahidol University, THAILAND

## Abstract

**Background:**

This systematic review aims to assess how different urbanisation patterns related to rapid urban growth, unplanned expansion, and human population density affect the establishment and distribution of *Aedes aegypti* and *Aedes albopictus* and create favourable conditions for the spread of dengue, chikungunya, and Zika viruses.

**Methods and findings:**

Following the Preferred Reporting Items for Systematic Reviews and Meta-Analyses (PRISMA) guidelines, a systematic review was conducted using the PubMed, Virtual Health Library, Cochrane, WHO Library Database (WHOLIS), Google Scholar, and and the Institutional Repository for Information Sharing (IRIS) databases. From a total of 523 identified studies, 86 were selected for further analysis, and 29 were finally analysed after applying all inclusion and exclusion criteria. The main explanatory variables used to associate urbanisation with epidemiological/entomological outcomes were the following: human population density, urban growth, artificial geographical space, urban construction, and urban density. Associated with the lack of a global definition of urbanisation, several studies provided their own definitions, which represents one of the study’s limitations. Results were based on 8 ecological studies/models, 8 entomological surveillance studies, 7 epidemiological surveillance studies, and 6 studies consisting of spatial and predictive models. According to their focus, studies were categorised into 2 main subgroups, namely “*Aedes* ecology” and “transmission dynamics.” There was a consistent association between urbanisation and the distribution and density of *Aedes* mosquitoes in 14 of the studies and a strong relationship between vector abundance and disease transmission in 18 studies. Human population density of more than 1,000 inhabitants per square kilometer was associated with increased levels of arboviral diseases in 15 of the studies.

**Conclusions:**

The use of different methods in the included studies highlights the interplay of multiple factors linking urbanisation with ecological, entomological, and epidemiological parameters and the need to consider a variety of these factors for designing effective public health approaches.

## Author summary

The expansion of urbanisation is often associated with the emergence and spread of vector-borne diseases by creating favourable conditions for the survival of *Aedes* species and the spread of dengue, chikungunya, and Zika viruses. This systematic review examined the relationship of urbanisation to the emergence and spread of *Aedes* mosquito–borne diseases and epidemics. From a total of 523 identified studies, 29 were included in the analysis. Studies were categorised into 2 main subgroups, namely “*Aedes* ecology” and “transmission dynamics” according to the main influence factors posed by urbanisation. Selected articles showed a clear relationship of urbanisation with distribution and density of *Aedes* mosquitoes and a robust association between vector production, human population density, and disease transmission. Differing definitions of ’urbanisation’ and the interplay of numerous factors linking urbanisation with ecological, entomological, and epidemiological parameters highlight the need for a multidimensional perspective when assessing the impacts of rapid and unplanned urban expansion and when designing effective control programmes.

## Introduction

Rapid unplanned urbanisation in combination with climate and environmental change, increased global travel and trade, and other societal challenges has stimulated the emergence or reemergence of vector-borne diseases [1–3]. Various studies have indicated that the intensification of urbanisation favours the spread of these diseases, which may also flourish due to a greater density of people as well as domestic and peridomestic animals [4,5].

According to the United Nations (UN) [6], urbanisation is the process whereby the proportion of a population living in urban areas and by which a large number of people become permanently concentrated in relatively small areas forming a city. It is estimated that 55% of the world’s population resided in urban areas in 2018, while by 2050, 68% of the world’s population is projected to be urban [7]. In addition, according to Bhatt and colleagues [8], areas where human population density is greater than or equal to 1,000 people per square kilometer are indicative of intense urbanisation, thus providing a suitable threshold for distinguishing urban from peri-urban and rural areas. According to Vlahov and colleagues [9], urbanisation refers to the change in size, density, and heterogeneity of cities, while factors such as population mobility, segregation, and industrialisation frequently accompany urbanisation. Thus, the process of urbanisation is not dependent on the definition of urban per se, but rather on the dynamics of agglomeration of individuals.

According to WHO [10], urbanisation is expected to bring about the emergence of new vector-borne diseases and further intensification of others, particularly viral diseases transmitted by *Aedes* mosquitoes. The control of *Aedes* species is rather complicated and requires the application of standardised control measures and quality control activities, monitoring protocols, community-based interventions, and emergency vector control plans to reduce the risk of an epidemic [11]. These interventions can prove particularly difficult to implement in cases of other escalating societal challenges, such as population movements and rapid and unplanned urbanisation [8,12].

The current study is based on preceding work that supports the hypothesis that urbanisation favours the spread of infectious diseases [2,8,13]. It attempts to contribute to previously conducted reviews on identified associations and gaps for studying the interrelationship of socioecological factors related to *Aedes* species and disease transmission in urban areas [14–16] by providing further evidence on how urbanisation patterns influence *Aedes* ecology and affect the spread and reemergence of *Aedes*-borne diseases.

Thus, the principal aim of this study was to evaluate the impact of urbanisation on the emergence and reemergence of *Aedes*-borne infectious diseases and the degree to which urbanisation processes create suitable habitats for *Aedes* mosquitoes. In particular, the study emphasises the following: (a) the influence of urbanisation on *Aedes* ecology; (b) the impact of urbanisation on disease transmission dynamics; (c) the role of human population density; and (d) the link between urbanisation and *Aedes*-borne diseases from a socioecological perspective.

## Materials and methods

This study follows the Preferred Reporting Items for Systematic Reviews and Meta-Analyses (PRISMA) guidelines for reporting systematic reviews and meta-analyses [17]. Preliminary searches were performed on PubMed to fine-tune the search protocol, and a review protocol was drawn up.

Terms used for the literature search were the following: “*Aedes aegypti*” or “*Aedes albopictus*” or “*Aedes*” and “dengue” or “chikungunya” or “Zika.” Searches were run with each one of these terms separately, or a set of them, in combination with “urbanisation” or “human population density.” The search was conducted using the appropriate Medical Subject Heading (MeSH) terms followed by the Boolean operator “OR” for terms within categories and “AND” between categories combined with “free text” terms. The terms used for the “vector” category included “*Aedes aegypti*” and “*Aedes albopictus*” and “*Aedes*.” The search terms for the “diseases” category included “dengue,” “chikungunya,” and “Zika.” These terms were used in different combinations together with the terms urbanisation and human population density in order to broaden the search.

The literature search was performed between February and April 2020 in the following databases: PubMed, Virtual Health Library, Cochrane, WHO Library Database (WHOLIS), Google Scholar, and the Institutional Repository for Information Sharing (IRIS). For searches in Google Scholar, we screened the first 200 hits and adding batches of a further 50 hits as long as the previous batch contained at least 1 relevant title. The search was initially conducted without any geographical, date, or language restriction in the selected databases by 2 independent reviewers (AK and SH). In a second step, articles were reviewed independently in full by these 2 reviewers, with a third reviewer available in case of disagreement between them. The references cited by each included article were also reviewed for potentially eligible studies.

Data extraction was carried out using a predefined data extraction sheet. Studies were first categorised into 4 groups according to study type: ecological studies or ecological models; entomological surveillance; epidemiological surveillance; and spatial and predictive models. The studies were further reviewed for the associated mosquito vector, either *Aedes albopictus* or *Aedes aegypti*, and reported diseases. All studies were then further analysed in regard to their urbanisation context as well as for their reported definition of urbanisation. It is worth noting that in most studies (*n* = 23), a separate definition for urbanisation was provided, thus highlighting (1) the lack of a universal definition for urbanisation; and (2) its dynamic and transformative aspect. In the remaining studies (*n* = 6), the effect of urbanisation was deduced mainly from the human population density criterion and confirmed through the overall urbanisation context of the study area.

Following further analysis of the measured outcomes and key findings, 5 main explanatory variables driving either the “*Aedes* ecology” or “disease transmission” were discerned. These variables were the following: (1) human population density; (2) urban growth; (3) artificial geographical space; (4) urban construction; and (5) urban density. The associations of these 5 explanatory variables with the 2 main factors influenced by urbanisation, “*Aedes* ecology” and “disease transmission,” were then presented in the evidence table (Table 1).

**Table 1:** Evidence table.

Author year	Study location and population density	Study type	Vector	Disease	Urbanisation variable	Measured outcome	Key findings and conclusions	Influence factor	Quality assessment score
*Aedes* ecology									
Estallo and colleagues (2018) [46]	Argentina, Cordoba; 2,273	Ecological model	*Aedes aegypti*	Dengue, chikungunya, and Zika	Urban construction	Environmental, socioeconomic, and demographic factors driving the distribution of *Ae*. *aegypti* larvae	The *Ae*. *aegypti* risk map showed a wider geographic distribution in areas with high human population density, as well as in places in which container habitats are available for egg laying and larval development, such as city channels, informal settlements abandoned train depots, and cemeteries	*Aedes* ecology	40
Fuentes-Vallejo and colleagues (2015) [47]	Colombia, Arauca; 4,321–9,332	Ecological model	*Ae*. *aegypti*	Dengue	Artificial geographic space	Relationship between the territory’s structures and dynamics and vector density	The study found a relationship between territorial structures and dynamics and vector density in both study areas, where the interaction between ecological and social systems shape areas with high and low *Ae*. *aegypti* density; higher density was related to unplanned urbanisation	*Aedes* ecology	100
Satoto and colleagues (2019) [25]	Indonesia, Magelang; 6,693	Entomological and epidemiological surveillance	*Ae*. *aegypti*	Dengue	Human population density and urban growth	Association between insecticide resistance and DHF case distribution related to human urbanisation	Study showed increasing population size and human urbanisation from less urban areas to urban areas in almost the same period as the occurrence of insecticide resistance, potentially related to slight increase in population size and human urbanisation	*Aedes* ecology	80
Zahouli and colleagues (2016) [20]	Côte d’Ivoire, Treichville; 1,800	Entomological surveillance	*Ae*. *aegypti*	Dengue and yellow fever	Human population density	Assess *Aedes* spp. oviposition ecology in variously urbanised settings	Urbanisation correlated with a substantially higher abundance in *Aedes* mosquitoes and a regression of the *Aedes* wild species towards a unique presence of *Ae*. *aegypti* in urban areas.	*Aedes* ecology	100
Li and colleagues (2014) [24]	China, Guangzhou; 3,000	Entomological surveillance	*Aedes albopictus*	Dengue	Human population density and urban growth	Determine how environmental changes due to urbanisation affect the ecology of *Ae*. *albopictus*	Urbanisation substantially increased the density, larval development rate, and adult survival time of *Ae*. *albopictus*, which, in turn, potentially increased the vector capacity, and, therefore, disease transmissibility	*Aedes* ecology	100
Zahouli and colleagues (2017) [21]	Côte d’Ivoire, Treichville; 1,800	Entomological surveillance	*Ae*. *aegypti*	Dengue and yellow fever	Human population density and urban growth	Larval ecology of *Aedes* mosquitoes in different settings (rural, suburban, and urban) in Côte d’Ivoire	In Côte d’Ivoire, urbanisation is associated with high abundance of *Aedes* larvae and a predominance of artificial containers as breeding sites, mostly colonised by *Ae*. *aegypti* in urban areas	*Aedes* ecology	100
Cox and colleagues (2007) [27]	Puerto Rico, San Juan; 3,187	Entomological surveillance	*Ae*. *aegypti*	Dengue	Urban construction	Investigate habitat distribution of adult *Ae*. *aegypti*	*Ae*. *aegypti* was positively associated with high-density housing and urban regions in urban and suburban areas of San Juan and was the dominant species in areas of high-density housing in urban areas	*Aedes* ecology	100
Manica and colleagues (2016) [23]	Italy, Rome; 6,000	Entomological surveillance and ecological model	*Ae*. *albopictus*	Dengue and chikungunya	Artificial geographic space and human population density	Ecoclimatic factors affecting *Ae*. *albopictus* abundance and dynamics in metropolitan versus suburban/rural sites in Rome (Italy), which was colonised by *Ae*. *albopictus* in 1997 and became one of the most infested metropolitan areas in Southern European temperate regions	*Ae*. *albopictus* presence found more abundant in strongly urbanised areas compared to more green ones; within metropolitan Rome, the highest densities were observed in small green areas within heavily urbanised settings	*Aedes* ecology	100
Samson and colleagues (2015) [22]	Haiti, Cap-Haïtien; 5,129	Entomological surveillance and ecological model	*Ae*. *albopictus* and *Ae*. *aegypti*	Dengue and chikungunya	Urban growth	Impact of unplanned urbanisation on mosquito ecology and vector-borne diseases by assessing land use and change patterns	*Ae*. *albopictus* and *Ae*. *aegypti* were collected more frequently from land use types categorised as urban and newly urbanised; rapid urbanisation following the 2010 earthquake increased the amount of area with suitable habitats for urban mosquitoes	*Aedes* ecology	100
Carbajo and colleagues (2006) [26]	Argentina, Buenos Aires; 15,000	Entomological surveillance and spatial model	*Ae*. *aegypti*	Dengue and yellow fever	Urban construction and human population density	Oviposition as a function of urbanisation variables	The proportion of weeks infested and the total number of eggs showed spatial continuity and were higher in areas that had higher densities of houses and were closer to industrial sites; the spatial pattern of *Ae*. *aegypti* oviposition in a highly urbanised city such as Buenos Aires was related to the urbanisation gradient	*Aedes* ecology	100
Ren and colleagues (2019) [35]	China, Guangzhou; 18,836	Spatial model	*Ae*. *albopictus*	Dengue	Human population density and urban growth	The relationships between reported DF epidemics during 2012–2017, GDP, the traffic system (road density, bus, and/or subway stations), and urban villages derived from high-resolution remotely sensed imagery in the central area of Guangzhou	Urban villages possessed higher values of DF cases density, incidence rates, and population density in the central region of Guangzhou City; urban villages result in a high environmental suitability for some vectors (e.g., *Ae*. *albopictus*), as well as the vector-borne diseases (i.e., DF) in these regions	*Aedes* ecology	100
*Aedes* ecology and disease transmission									
Messina and colleagues (2019) [36]	Global	Ecological model	*Ae*. *aegypti* and *Ae*. *albopictus*	Dengue	Human population density and urban density	Climate, population, and socioeconomic projections for the years 2020, 2050, and 2080 to project future changes in virus suitability and human population at risk	DENV transmission is maintained in urban settings where humans and mosquitoes are the only known hosts, with a sylvatic cycle occurring in nonhuman primates in forested areas and rarely resulting in transmission to humans	*Aedes* ecology and disease transmission	100
Piedrahita and colleagues (2018) [29]	Colombia, Medellin; 6,925	Epidemiological surveillance and spatial analysis	*Ae*. *aegypti*	Dengue	Human population density	Impact of population density and entomological indexes associated with the spatial distribution of DENV seroprevalence	Population density and *Ae*. *aegypti* house index were significantly correlated with the observed pattern; high DENV risk was found in districts with combined poorly socioeconomic conditions and densest human and mosquito populations	*Aedes* ecology and disease transmission	60
Padmanabha and colleagues (2012) [37]	Colombia, Armenia; 2,100	Spatial model	*Ae*. *aegypti*	Dengue	Human population density	Evaluate the combined impacts of variation in *Ae*. *aegypti* production and human density	Increased human density favoured Dengue R0, and when the likelihood of human introduction of virus was incorporated into risk, a strong interaction arose between vector production and human density	*Aedes* ecology and disease transmission	80
Disease transmission									
Freitas and colleagues (2019) [38]	Brazil, Rio De Janeiro; 5,249	Ecological	*Ae*. *aegypti* and *Ae*. *albopictus*	Dengue, chikungunya, and Zika	Human population density and artificial geographic space	Detect spatiotemporal clustering for each disease separately and for all 3 simultaneously	Simultaneous clusters of the 3 diseases were more likely in neighbourhoods with a combination of high population density and low socioeconomic status	Disease transmission	80
Delmelle and colleagues (2016) [45]	Colombia, Cali; 4,000	Ecological model	*Ae*. *aegypti* and *Ae*. *albopictus*	Dengue	Human population density	The dynamics of DF transmission relevant to changes in environmental conditions, as well as local demographic and socioeconomic factors	Among the strongest predicting variables for DF were population density and socioeconomic stratum	Disease transmission	100
Bouzid and colleagues (2014) [39]	Europe and Mexico	Ecological model	*Ae*. *albopictus* and *Ae*. *aegypti*	Dengue	Human population density	Dengue risk in Europe under climate change scenarios; DF risk in Europe in terms of disease occurrence rather than mosquito presence	Population and urbanisation projections for Europe show that minor changes are expected (with some local variation), especially when compared to other parts of the world (where significant increase in population size and urbanisation are expected until 2100); climate change is likely to contribute to increased dengue risk (and possibly other mosquito-borne diseases) in many parts of Europe, especially towards the end of the century	Disease transmission	100
Cao and colleagues (2017) [40]	China, Guangzhou; 17,562	Ecological model	*Ae*. *albopictus*	Dengue	Human population density and urban construction	Investigate the independent and interactive effects of several socioecological factors on the 2014 dengue epidemic at a township/street level in Guangzhou	Socioecological factors including road density, temperature, urbanisation level, and urban village, might either separately or jointly influence the spatial distribution of DF in Guangzhou	Disease transmission	100
Akhtar and colleagues (2016) [48]	India, Delhi, 11,312	Ecological model	*Ae*. *aegypti*	Dengue	Urban growth and urban density	Link of dengue prevalence with the heat entrapped by the urban structure accentuating the temperature and humidity, thus helping *Aedes* mosquitoes to breed faster and in larger number	Correlation was found of urban density to total cases and urban population to total cases; seasonal or cyclic factors of the disease are combined with the fluctuating humidity and temperature data and the urban density (a proxy variable of urban growth) that creates heat effect in some of the dense area and thus leads to pocketed outbreak of the disease	Disease transmission	40
Teixeira and colleagues (2007) [28]	Brazil, Salvador; 1,834–49,980	Epidemiological surveillance	*Ae*. *aegypti*	Dengue	Human population density	Relationship between the intensity of virus circulation and the population’s living conditions or between group immunity and *Ae*. *aegypti* infestation rates	The risk of infection was high in almost all the areas, including in the areas with good living conditions. It is likely that these dynamics is at least partially due to the fact that in Salvador, high population density is found both in areas with precarious living conditions and in those where economically more favoured populations live	Disease transmission	100
Qi and colleagues (2015) [34]	China, Pearl River Delta; 8,687	Epidemiological surveillance and ecological model	*Ae*. *albopictus*	Dengue	Human population density and urban growth	Assess core contributors to the occurrence of DF from the perspective of the social economy and the environment	DF transmission has been reported in both rural and urban areas. However, urban environments are characterised by many factors, such as a higher population, poor hygiene, poor housing conditions, and less environmental management; rapid urbanisation with large populations living in peri-urban slums provides attractive features for the *Aedes* mosquito and promotes DF transmission	Disease transmission	100
Barrera and colleagues (2000) [30]	Venezuela, Maracay; 1,439	Epidemiological surveillance and spatial analysis	*Ae*. *aegypti*	Dengue	Human population density	Relation of dengue with the number of inhabitants and population density, during the 1993–1998 period, leading to 10,576 reported cases of dengue, 2,593 cases of DHF and 8 deaths	The incidence of DHF was significantly related to the incidence of dengue, the number of inhabitants in an area, and population density	Disease transmission	100
Vallée and colleagues (2009) [33]	Laos, Vientiane; 3,255	Epidemiological surveillance and spatial analysis	*Ae*. *aegypti* and *Ae*. *albopictus*	Dengue	Human population density and urban construction	Explore the link between flavivirus seroprevalence and urbanisation levels of residential neighbourhoods	Level of urbanisation and length of residence were the 2 most significant factors in predicting individual risk of flavivirus infection; significant association between flavivirus seroprevalence and urbanisation was found within Vientiane City	Disease transmission	100
Wu and colleagues (2009) [31]	Taiwan; 7,100	Epidemiological surveillance and spatial analysis	*Ae*. *aegypti* and *Ae*. *albopictus*	Dengue	Human population density	Role of urbanisation and temperature increase in spatial patterns of dengue	Higher level of urbanisation was also associated with increasing risk on the occurrence of DF at township level; changing temperature pattern and urbanisation as most important determinants predicting DF occurrence in Taiwan	Disease transmission	100
Lin and colleagues (2011) [32]	Taiwan; 7,100	Epidemiological surveillance and spatial model	*Ae*. *aegypti* and *Ae*. *albopictus*	Dengue	Human population density	Local spatial variations of dengue–mosquito and dengue–human relationships within a study area	Higher human densities were shown to contribute to higher dengue incidence rates; in some areas, higher dengue incidences were associated with higher vector/host densities, but in some areas, higher incidences were related to lower vector/host densities	Disease transmission	80
Struchiner and colleagues (2015) [44]	Singapore; 7,804	Predictive model	*Ae*. *aegypti*	Dengue	Human population density	Contributions of putative drivers for the rise of dengue in Singapore: population growth, climate parameters, and international air passenger arrivals from dengue endemic countries, for the time period of 1974 until 2011	Population growth was the leading independent factor associated with the increase in dengue cases observed in Singapore over the past 40 years, followed by mean temperature change	Disease transmission	100
Zheng and colleagues (2019) [41]	China, Pearl River Delta; 6,000	Spatial model	*Ae*. *aegypti* and *Ae*. *albopictus*	Dengue	Human population density and urban density	Spatiotemporal patterns and potential influencing factors of DF epidemics	Population density and urban land ratio were the socioeconomic factors explaining the largest variance in regional epidemics in terms of spatial distribution	Disease transmission	100
Yue and colleagues (2018) [42]	China, Guangdong; 1,928	Spatial model	*Ae*. *albopictus* and *Ae*. *aegypti*	Dengue	Human population density	Environmental and socioeconomic risk factors leading to DF	DF was positively correlated with population density and GDP in study areas; water and a suitable temperature are also essential factors for the larvae of the DENV vector, the *Aedes* mosquito	Disease transmission	100
Khalid and colleagues (2015) [43]	Pakistan, Rawalpindi: 8,100; Islamabad: 2,089; Lahore: 6,300; Karachi: 3,900	Spatial model	*Ae*. *aegypti*	Dengue	Urban growth, urban density, and human population density	How change in urbanisation and population density and meteorological parameters affect dengue transmission	Increased urbanisation and population density has supported the dengue transmission in the mega cities of Pakistan. The anthropogenic activities have the great influence on the dengue transmission, survival, and growth as it require the urban environments. Increased urbanisation is associated with the increased number of dengue cases in study areas particularly in Lahore District, which is supported by the natural topographic features with dengue supporting environments developed by human settlements and population density.	Disease transmission	80

DENV, dengue virus; DF, dengue fever; DHF, dengue hemorrhagic fever; GDP, gross domestic product.

The preestablished inclusion criteria comprised (i) studies examining how/if urbanisation affects the density, larval development rate, breeding sites, and adult survival time of *Ae*. *albopictus* and *Ae*. *aegypti*; (ii) studies examining how/if urbanisation affects the establishment, distribution, and vector density of *Ae*. *albopictus* and *Ae*. *aegypti*; (iii) studies examining how/if human population density favours the spread of dengue, Zika, and chikungunya incidence cases and epidemics; (iv) studies examining how/if geospatial patterns of urbanisation or human population density affect the seroprevalence and incidence of *Aedes* borne diseases; and (v) studies examining how/if the urbanisation trends create a syndemic effect that favours the incidence of dengue, chikungunya, or Zika.

Exclusion criteria were (i) studies not including urbanisation or human population density as a potential predictor variable or an explanatory variable for the incidence of dengue, chikungunya, or Zika or for *Aedes* population dispersal; (ii) studies focusing on mosquito species other than *Ae*. *albopictus* or *Ae*. *Aegypti*; (iii) studies focusing on Knowledge, Attitudes, and Practices (KAP) methodologies or clinical and laboratory characteristics with minor reference to urbanisation or human population density; (iv) studies conducted in urbanised settings consisting of a human population density less than 1,000 inhabitants per square kilometer; and (v) opinion papers or descriptive studies not providing numerical values or correlation indexes.

### Quality assessment

The variety of methodological approaches taken by the studies included in the current analysis justified the use of a mixed methods tool for appraising different qualitative and quantitative aspects of the included studies. The Mixed Methods Appraisal Tool (MMAT) was selected for its potential to assess the methodological quality of quantitative, qualitative, and mixed methods studies [18,19]. A total score based on a percentage scale was calculated for each study by dividing the number of criteria met by the number of criteria assessed (15 for mixed methods studies and 5 for other studies). For each criterion met, a “Yes” value was given, in contrast to “No” or “Can’t tell” in case of not meeting the criterion. Studies ranged from 0% (zero quality) through 20% (very low quality), 40% (low quality), 60% (moderate quality), 80% (considerable/good quality) to 100% (very high quality). About half of the studies (*n* = 15) were assessed on the mixed methods studies’ criteria, while the rest of the studies (*n* = 14) were assessed for their qualitative criteria. All studies were assessed by 2 reviewers (AK and SH). The quality assessment was not used for further exclusion of studies, but was considered in the interpretation of results.

## Results

### Study selection

**Fig 1:**
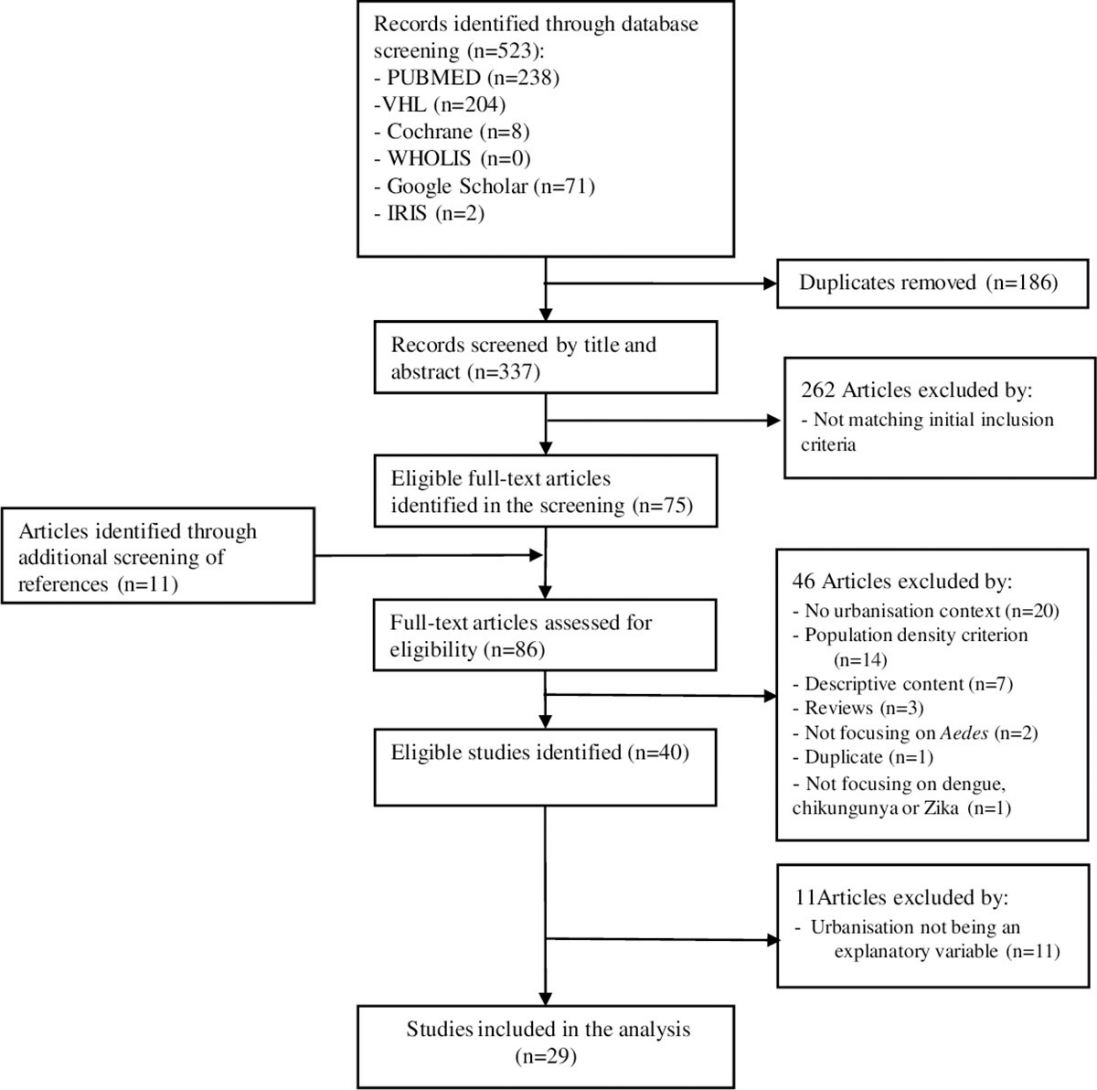
PRISMA flow diagram. IRIS, Institutional Repository for Information Sharing; PRISMA, Preferred Reporting Items for Systematic Reviews and Meta-Analyses; VHL, Virtual Health Library; WHOLIS, WHO Library Database.

A total of 523 articles with abstracts were identified through the database searches. Following the removal of 186 duplicates, 337 records were initially screened based on title and abstract by 2 reviewers (AK and SH). A total of 86 full-text articles were assessed further for eligibility, 11 of which were identified through additional screening of reference lists. In total, 29 articles [20–48], were included in the systematic review (Fig 1).

### General study characteristics

All 29 studies were published between 2000 and 2019: A total of 12 were conducted in Asia, 11 in the Americas, 2 in Africa, 2 in Europe, and 2 consisted of modelling studies conducted in wider geographical contexts. The studies were further allocated into 4 principal groups according to their main study type: 8 studies were ecological studies or ecological models; another 8 studies were based on entomological surveillance methods; 7 studies employed epidemiological surveillance methods; and 6 were grouped as spatial and predictive models. It should be noted that 10 of the studies used a mixture of at least 2 of the 4 categorised study types. Studies were divided into 2 subgroups according to the main influence factor posed by urbanisation, “*Aedes* ecology” and “disease transmission” (Table 1). More than half (*n* = 15) of the studies focused on “disease transmission,” 11 studies on the impact of urbanisation on *Aedes* ecology, and 3 studies on a combination of both factors (Table 1). *Ae*. *aegypti* was identified as the main vector of interest in most of the studies (*n* = 14), and 5 studies were mainly concerned with *Ae*. *albopictus*, while in the rest of the studies (*n* = 10) reference was made to both species. Dengue was the main disease of interest in most of the studies (*n* = 25), while chikungunya and Zika were mentioned in combination with dengue in 4 studies. The majority of studies (*n* = 26) obtained quality assessment (MMAT) scores corresponding to “good” to “very high” quality, but 2 were rated as “low quality,” and 1 study as being of “moderate quality.”

### Evaluation of the findings

#### Influence of urbanisation on *Aedes* ecology

The expansion of urban development appears to play a significant role in the distribution of the *Aedes* population. Several studies [20–27] used entomological surveillance methods based on adult and larval sampling methods which, in several cases, were included in a spatial analysis [22,23,25,26]. Two studies conducted in the Ivory Coast [20,21] employing standard ovitrap methods showed a correlation of urbanisation with a substantially higher abundance of ovipositing *Aedes* mosquitoes. In particular, the numbers of emerged adult *Ae*. *aegypti* were higher in the urban (1.97 *Aedes*/ovitrap/week) than in the suburban (1.44 *Aedes*/ovitrap/week) and the rural (0.89 *Aedes*/ovitrap/week) areas [20]. Also, proportionally, more potential breeding sites were found to be utilised in urban (2,136/3,374, 63.3%) compared to suburban (1,428/3,069, 46.5%) and rural areas (738/2,423, 30.5%) [21]. Similar findings were described in a study conducted in Haiti, which used random mosquito larvae sampling and land use classifications to build a model of spatial distribution [22]. Due to an increase of suitable and partially new larval habitats, *Ae*. *albopictus* and *Ae*. *aegypti* were collected more frequently from land use types categorised as urban and as newly urbanised after an earthquake in 2010 [22].

Manica and colleagues [23] employed longitudinal adult monitoring combined with generalised linear mixed models and generalised additive mixed models to study ecoclimatic factors affecting *Ae*. *albopictus* abundance and dynamics in metropolitan versus suburban/rural sites in Rome (Italy). They found that high adult abundance was on average associated with highly anthropised habitats (rather than with highly vegetated ones) in both the metropolitan and the suburban/rural areas [23]. This is consistent with characteristics of highly anthropised habitats that favour the mosquito life cycle, such as high human population density providing more opportunities for blood feeding and larger numbers of artificial water containers (as for instance flowerpots, rain catch basins, abandoned tyres, and discarded tins). This within-setting feature of a gradient of *Aedes* abundance across a gradient of urbanisation levels was fairly common among the studies. In Indonesia, Satoto and colleagues [25], using ovitrap sampling methods, showed increasing trends of *Ae*. *aegypti* population size from less urban areas to urban areas.

In Buenos Aires (Argentina), Carbajo and colleagues [26] showed that the proportion of weeks infested (a trap was considered to be infested in a week when at least 1 egg was found) and the total number of eggs followed spatial continuity and were higher in areas that had higher densities of houses and that were closer to industrial sites. Cox and colleagues [27] employed a container mosquito sampling method to investigate habitat distribution of adult mosquitoes and found that *Ae*. *aegypti* was significantly associated (*p* < 0.05) with high-density housing in urban and suburban areas of San Juan (Puerto Rico) based on a canonical correspondence analysis.

For Guangzhou (China), Li and colleagues [24] showed that urbanisation substantially increased the mosquito density (adults), larval development rate, and adult survival time of *Ae*. *albopictus*, which, in turn, potentially increased the vector capacity, and, therefore, arbovirus transmissibility.

#### Impact of urbanisation on disease transmission dynamics

Many of the studies employed epidemiological surveillance methods to analyse the seroprevalence and incidence of *Aedes-*borne diseases in different urbanisation contexts.

In Salvador (Brazil) [28], dengue seroprevalence presented a positive correlation (*r* = 0.49; *p* = 0.006) with human population density in all study areas regardless of their different socioeconomic levels. In a study conducted in Colombia [29], a longitudinal serological survey was combined with spatial analysis, showing that human population density and *Ae*. *aegypti* house index were significantly correlated with the observed dengue immunoglobulin G (IgG) seroprevalence. Geostatistical regression analysis showed that dengue IgG seroprevalence was clustered, and 40% of the pattern observed arose out of districts with crowded population density (adjusted *R*^*2*^ = 0.38; *p* < 0.01) and a higher *Ae*. *aegypti* house index (adjusted *R*^*2*^ = 0.28; *p* < 0.05) [29]. In another study conducted in Venezuela [30], which also combined a longitudinal serological survey (IgG) with spatial analysis, a direct and significant relationship of registered dengue cases was found with human population density in 7 metropolitan municipalities of Maracay city (*r* = 0.94, *p* < 0.01).

Two studies conducted in Taiwan [31,32] combined longitudinal serological surveys with spatial analyses and found a significant correlation of both dengue IgG seroprevalence and dengue haemorrhagic fever incidence with human population density. One of the studies also found a significant positive association between cumulative incidence, numbers of months with average temperature above 18°C, and urbanisation (*R*^*2*^ = 0.135), while urbanisation was positively associated with the distribution of dengue incidence in a spatial lag model (*R*^*2*^ = 0.433) [31]. Combining a randomised cross-sectional serological survey with spatial analysis, Vallée and colleagues (2009) [33] found a significant association (*p* < 0.001) between previous dengue flavivirus infections and urbanisation in the city of Vientiane in Laos. More precisely, the prevalence of previous flavivirus infections was significantly (*p* < 0.001) higher in the central zone (60.1%; 95% confidence interval [CI] = 56.2 to 64.1) than in the first (51.1%; 95% CI = 48.7 to 53.5) and second (44.3%; 95% CI = 41.5 to 47.2) urbanised belts (periphery).

Qi and colleagues [34], employing a generalised additive model in the Pearl River Delta (China), found that urban areas, higher road density, and lower gross domestic product (GDP) per capita were consistent risk factors for dengue fever outbreaks. They found that rapid urbanisation with large populations living in peri-urban slums provided attractive features for the *Aedes* mosquito and promoted dengue transmission.

#### The role of human population density

Human population density is the leading metric used in the reviewed studies to differentiate urban from suburban/rural areas [20,21,24–26,35,36], and it is regularly used as an explanatory variable for levels of arboviral diseases [29–35,37–44].

Ren and colleagues [35] examined the association between dengue and high population density in newly formed “urban villages” in the central region of Guangzhou (China), showing that these villages form hubs of high environmental suitability for vectors (e.g., *Ae*. *albopictus*) as well as for the spread of dengue. Approximately 90% of total dengue cases were concentrated in these urban villages, while the number of cases was positively associated with acreage of the urban villages (*r* = 0.45, *p* = 0.015) [35]. In another study conducted in the Pearl River Delta and the Chinese border of Yunnan and Myanmar, investigating the spatiotemporal characteristics and primary influencing factors of typical dengue epidemics in China, Zheng and colleagues [41] found that population density and urban land ratio were the socioeconomic factors explaining the largest variance (>54%) in regional epidemics. Yue and colleagues [42] also found that the number of dengue fever cases was positively correlated with population density (*r* = 0.705) in Guangdong (China), while the presence of water and a suitable temperature were also essential factors for the larvae of the dengue virus vectors *Ae*. *albopictus* and *Ae*. *aegypti*. Higher human population density along with temperature patterns were also identified as the most important determinants of dengue incidence rates in studies conducted in Singapore [44] and Taiwan [31,32].

Delmelle and colleagues [45] in a study conducted in Cali (Colombia) identified human population density as one of the risk factors significantly contributing to the 2010 dengue fever outbreak (*R*^*2*^ = 0.295), while a strong correlation was found between relative human population density and socioeconomic stratum (*r* = −0.541, *p* < 0.01). In another study in Armenia (Colombia) [37], results indicated that increased human density favoured a greater average number of secondary infections with dengue (higher R_0_) through both human-to-mosquito and mosquito-to-human transmission. A significant interaction was observed between human density and *Ae*. *aegypti* super-production, indicating that removal of “super-production” spots in areas of higher human density can have a high impact on reducing dengue [37].

#### Link between urbanisation and *Aedes*-borne diseases from a socioecological perspective in ecological studies and models

Numerous climatic, environmental, and social factors associated with urbanisation were included in several socioecological or modelling studies to explain the spread of *Aedes-*borne diseases.

Messina and colleagues [36] used an ecological niche model to investigate climate, population, and socioeconomic projections for the years 2020, 2050, and 2080 to predict future changes in transmission suitability and the size of the human population at risk. Their model also incorporated information about the spread of *Aedes* vectors, urbanisation, and population growth [36]. They predicted that approximately 2.25 billion more people will be at risk of dengue in 2080 compared to 2015, bringing the total population at risk to over 6.1 billion. Similarly to the previous study, Bouzid and colleagues [39] showed in a general additive model using data from Mexico and Europe that urbanisation and population are significantly associated with an increased risk of dengue incidence and that climate change is likely to contribute to increased dengue risk (and possibly other mosquito-borne diseases) in many parts of Europe, especially towards the end of the century.

Freitas and colleagues [38] pointed out in a study conducted in Rio de Janeiro (Brazil) that simultaneous clusters of dengue, chikungunya, and Zika viruses were more likely in neighbourhoods with a combination of high human population density and low socioeconomic status. Estallo and colleagues [46] used ecological niche modelling to show the risk of a wider geographic distribution of *Ae*. *aegypti* in areas of Córdoba (Argentina), while Fuentes Vallejo and colleagues [47] employed a chorematic model in Arauca (Colombia). The latter found a relationship between territorial structures, their dynamics, and vector density [47]. Another finding of this study was that the interaction between ecological and social systems forms areas with high and low *Ae*. *aegypti* density, with a higher density being related to unplanned urbanisation [47].

Cao and colleagues [40] investigated the independent and interactive effects of several socioecological factors including road density, temperature, and urbanisation level on the 2014 dengue epidemic at a township/street level in Guangzhou (China). Dengue fever incidence was positively correlated with the human population density (*r* = 0.49, *p* < 0.01), road density (*r* = 0.36, *p* < 0.01), urbanisation level (*r* = 0.42, *p* < 0.01), and the geographical ratio of urban villages (extent of high-density and low-rise building areas) (*r* = 0.28, *p* < 0.01) [40]. In a study examining the link between dengue prevalence and the heat entrapped by the urban structure of Delhi (India), Akhtar and colleagues [48] found that the mean annual temperature shows significant positive relationship with the urbanisation variables—urban population (*r* = 0.539), urban density (*r* = 0.539), and vehicular population (*r* = 0.405). Their study is able to explain 57% of the total variation and shows association between disease prevalence, urbanisation, and climatic factors. In addition, seasonal or cyclical factors of the disease were combined with the fluctuating humidity and temperature data and the urban density (a proxy variable of urban growth), showing that the heat effect in some of the dense areas may lead to pocketed outbreaks of the disease [48]. Khalid and colleagues [43] employed a geospatial model to examine how changes in urbanisation, human population density, and meteorological parameters affect dengue transmission in 4 megacities of Pakistan. A heightened number of dengue cases is associated with increased urbanisation and is also enhanced by natural topographical elements as well as environments that support dengue, such as human settlements and high population density [43].

## Discussion

Urban environments create favourable conditions for disease transmission in which people are increasingly densely arranged in complex multistory built environments linked via global flows of travellers and goods that complicate public health responses [49]. Urbanisation is a dynamic phenomenon resulting in “megacities” with high population density, while it leads at the same time to new spatial landscapes often characterised by insufficient infrastructure and services, thus creating ideal conditions for increased mosquito-, rodent-, and water- and food-borne infectious diseases [13]. Urbanised areas can be characterised as “coupled human natural systems,” leading to an imperative need to explain patterns of disease emergence in relation to urbanisation from a socioecological perspective [13].

The current study highlights consistent conclusions concerning the impact of urbanisation on both *Aedes* ecology and disease transmission dynamics:

- Urbanisation (defined by population density or by artificial geographical space) correlates with a significantly higher risk and abundance of *Aedes* mosquitoes through provision of favourable breeding sites, higher larval development rate, and adult survival time.
- The degree of urbanisation and population density is significantly associated with a consistent gradient in disease incidence.
- Rapid urbanisation with large populations living in unplanned urban areas provides attractive features for the *Aedes* mosquito and promotes disease transmission.
- Socioecological factors might either separately or jointly influence the spatial distribution of *Aedes* mosquitoes and disease transmission.

The variety of methodological approaches taken in the included articles mirrors the interplay of multiple factors linking urbanisation with ecological, entomological, and epidemiological aspects. It also calls for a multifaceted approach in designing effective public health interventions. Reliance on single control tools has historically failed to sustainably control these invasive mosquitoes or the pathogens they transmit [50]. Targeted interventions should be guided by informed public planning and education programmes, weighted by the investment capacity of each case area, bearing in mind that vector control techniques are not always easy to implement in urbanised settings because of the species’ ability to develop in a wide range of artificial breeding sites [10].

The risk of increased disease transmission in urban contexts requires the enhancement of active public health surveillance programmes assisted by modern laboratory virology for effective tracking of dengue and other emerging arboviral diseases. Supported also by the evidence of the current study, specific emphasis should be placed on the association of urbanisation and dengue. Based on the spatiotemporal distribution of available seroprevalence studies worldwide, most of them were conducted on dengue (66.5%), while 16% were exclusively conducted on chikungunya, and only 7% were exclusively concerned with Zika [51]. In addition, the spatial risk profiling of dengue transmission is necessary to ensure the optimal utilisation of resources and achievement of maximum impact of vector control [52]. It is noteworthy that most of the selected articles provide their own definitions of urbanisation. This reflects the numerous aspects associated with urbanisation and the emergence and spread of *Aedes* populations and arboviruses. In many studies [22,23,25,26,29–32,35–42,43,45–48], urbanisation patterns were identified through the use of satellite images and geospatial analysis detecting territorial classifications mainly relevant to land use change, urban construction, and road density of study areas. In several studies [22,34,35,40,43–45,47,48], urbanisation was characterised by rapid unplanned development accompanied by poor sanitation, overcrowding population, and deficient infrastructure, thus favouring the spread of *Aedes* mosquitoes and the pathogens they transmit.

The main limitation of the current study is associated with the validity (risk of bias) of the included studies. Specifically, urbanisation is often defined through the use of a simple dichotomy, urban versus rural, or even a single continuous variable such as human population density. This indicates a substantial inconsistency, which hampers our understanding of the specific changes within the process of urbanisation that affect risk and disease. The concept of a “threshold” delineating an urban area as opposed to other types of living environment ignores the graduation in disease states reported between and within geographical areas and misses the variation and dynamism that underlies these populations [53].

Considering the aforementioned limitations and the absence of a global standard to delineate cities, urban, and rural areas for international statistical comparisons [54], the criteria used in the current study to define urbanisation and human population density were reviewed independently for each article regarding their quality and according to the criteria laid down during the stage of study selection. This approach aimed to clearly articulate the qualitative and dynamic aspects of urbanisation relevant to the study’s objectives.

## Conclusions

The findings of this systematic review contribute to the discourse on public health challenges posed by urbanisation concerning the spread of *Aedes*-borne diseases in areas mainly characterised by high population density or rapid and unplanned urbanisation patterns. The disease dynamics in urban contexts pose a significant threat for public health systems and require multisectoral and multidisciplinary approaches for the design and application of informed prevention and control strategies. It becomes evident that a growing number of areas around the globe are becoming increasingly vulnerable to the spread of vectors and diseases boosted by rapid unplanned urbanisation in combination with other socioecological challenges including climate and environmental change [55,56]. The increasing representation of urbanised populations in future projections highlights the pressing need for an improved disentangling of the numerous phenomena linking cities with arbovirus transmission [57].

## Supporting information

S1 Table Literature search for all databases. (DOCX)

S2 Table Quality assessment. (XLSX)

S3 Table Data extraction table. (XLSX)

S4 Table PRISMA checklist. PRISMA, Preferred Reporting Items for Systematic Reviews and Meta-Analyses.
(DOCX)

S1 File PubMed example search. (XLSX)

S2 File Review protocol. (PDF)
