# Supplementary material for: The role of urbanisation in the spread of Aedes mosquitoes and the diseases they transmit—A systematic review
Source: PLoS Negl Trop Dis. 2021 Sep 9;15(9):e0009631. doi: 10.1371/journal.pntd.0009631 (PMC8428665; doi:10.1371/journal.pntd.0009631)
Supplement: S1 Table — (DOCX) [file pntd.0009631.s001.docx]

| Number | 1. Search term | 2. Search term | 3. Search term |
| --- | --- | --- | --- |
| **1** | Aedes Aegypti OR  Aedes Albopictus | Dengue OR  Chikungunya OR ZIKA | Urbanization |
| 2 | N/A | Dengue | Urbanization |
| 3 | Aedes Aegypti OR  Aedes Albopictus | Dengue OR  Chikungunya OR ZIKA | Human Population Density |
| 4 | N/A | Dengue | Human Population Density |
| 5 | N/A | Chikungunya | Urbanization |
| 6 | N/A | Zika | Urbanization |
| 7 | Aedes | N/A | Urbanization |

**PUBMED**

**Virtual Health Library**

| Number | 1. Search term | 2. Search term | 3. Search term |
| --- | --- | --- | --- |
| **1** | aedes | Dengue OR  Chikungunya OR ZIKA | Urbanization |
| 2 | N/A | Dengue | Urbanization |
| 3 | aedes | Dengue OR  Chikungunya OR ZIKA | Human Population Density |
| 4 | N/A | Dengue | Human Population Density |
| 5 | N/A | Chikungunya  (NOT DENGUE NOT ZIKA) | Urbanization |
| 6 | N/A | Zika | Urbanization |
| 7 | Aedes | N/A | Urbanization |

**Cochrane**

| Number | 1. Search term | 2. Search term | 3. Search term |
| --- | --- | --- | --- |
| **1** | Aedes | Dengue OR  Chikungunya OR ZIKA | Urbanization |
| 2 | N/A | Dengue | Urbanization |
| 3 | Aedes | Dengue OR  Chikungunya OR ZIKA | Human Population Density |
| 4 | N/A | Dengue | Human Population Density |

**WHOLIS**

| Number | 1. Search term | 2. Search term | 3. Search term |
| --- | --- | --- | --- |
| **1** | Aedes OR  Aedes Aegypti OR  Aedes Albopictus | Dengue OR  Chikungunya OR ZIKA | Urbanization |
| 2 | N/A | Dengue | Urbanization |
| 3 | Aedes OR  Aedes Aegypti OR  Aedes Albopictus | Dengue OR  Chikungunya OR ZIKA | Human Population Density |
| 4 | N/A | Dengue | Human Population Density |

**IRIS**

| Number | 1. Search term | 2. Search term | 3. Search term |
| --- | --- | --- | --- |
| **1** | Aedes | Dengue OR  Chikungunya OR ZIKA | Urbanization |
| 2 | N/A | Dengue | Urbanization |
| 3 | Aedes | Dengue OR  Chikungunya OR ZIKA | Human Population Density |
| 4 | N/A | Dengue | Human Population Density |

| Number | 1. Search term | 2. Search term | 3. Search term |
| --- | --- | --- | --- |
| **1** | aedes | Dengue OR  Chikungunya OR ZIKA | Urbanization |
| 2 | N/A | Dengue | Urbanization |
| 3 | aedes | Dengue OR  Chikungunya OR ZIKA | Human Population Density |
| 4 | n/a | Dengue | Human Population Density |
| 5 | Aedes | N/A | Urbanization |

**Google Scholar**

**PUBMED**

| Number | 1. Search term | 2. Search term | 3. Search term | Hits | Records Screened |
| --- | --- | --- | --- | --- | --- |
| **1** | Aedes Aegypti OR  Aedes Albopictus | Dengue OR  Chikungunya OR ZIKA | Urbanization | 886 | 141 |
| 2 | N/A | Dengue | Urbanization | 1351 | 23 |
| 3 | Aedes Aegypti OR  Aedes Albopictus | Dengue OR  Chikungunya OR ZIKA | Human Population Density | 427 | 48 |
| 4 | N/A | Dengue | Human Population Density | 546 | 7 |
| 5 | N/A | Chikungunya | Urbanization | 0 | 0 |
| 6 | N/A | Zika | Urbanization | 0 | 0 |
| 7 | Aedes | N/A | Urbanization | 1351 | 19 |
