# Supplementary material for: The role of urbanisation in the spread of Aedes mosquitoes and the diseases they transmit—A systematic review
Source: PLoS Negl Trop Dis. 2021 Sep 9;15(9):e0009631. doi: 10.1371/journal.pntd.0009631 (PMC8428665; doi:10.1371/journal.pntd.0009631)
Supplement: S2 File — (PDF) [file pntd.0009631.s006.pdf]

Review Protocol

Review title

The role of urbanisation in the spread of *Aedes*-borne diseases - A systematic review

Anticipated or actual start date
Give the date when the systematic review commenced, or is expected to commence.
15/02/2020

Anticipated completion date
Give the date by which the review is expected to be completed.
30/11/2020

Named contact
Olaf Horstick

Named contact email
Olaf.Horstick@uni-heidelberg.de

Review team members and their organisational affiliations

Dr Olaf Horstick, Heidelberg Institute of Global Health, Research to Practice Group, Heidelberg University, Germany

Dr Antonios Kolimenakis, Benaki Phytopathological Institute, Scientific Directorate of Entomology and Agricultural Zoology, Greece

Mrs Sabine Heinz, Heidelberg Institute of Global Health, Research to Practice Group, Heidelberg University, Germany

Dr Michael Lowery Wilson, Heidelberg Institute of Global Health, Research to Practice Group, Heidelberg University, Germany

Dr Volker Winkler, Heidelberg Institute of Global Health, Research to Practice Group, Heidelberg University, Germany

Dr Laith Yakob, London School of Hygiene and Tropical Medicine, London, United Kingdom

Dr Antonios Michaelakis, Benaki Phytopathological Institute, Scientific Directorate of Entomology and Agricultural Zoology, Greece

Dr Dimitrios Papachristo, Benaki Phytopathological Institute, Scientific Directorate of Entomology and Agricultural Zoology, Greece

Dr Clive Richardson, Panteion University of Social and Political Sciences, Department of Economic and Regional Development, Greece

Funding sources/sponsors

This research is co-financed by Greece and the European Union (European Social Fund- ESF) through the Operational Programme «Human Resources Development, Education and Lifelong Learning» in the context of the project “Reinforcement of Postdoctoral Researchers - 2nd Cycle” (MIS-5033021), implemented by the State
Scholarships Foundation (IKY)."

Conflicts of interest

None known

Review question(s)

a) how urbanization and its spatial characteristics affect the establishment and distribution of Aedes albopictus or Aedes aegypti and their potential vectorial capacity to transmit dengue chikungunya and zika viruses;
b) how human population density and conglomeration of human population in urban areas favor the spread of Aedes species and their associated diseases and epidemics;
c) how urbanization dynamics create complex landscapes influencing the Aedes distribution and their associated diseases incidence.

Searches

The literature search will be performed in the following databases: PubMed, Virtual Health Library, Cochrane, WHOLIS, Google Scholar and IRIS. The search will be conducted without any geographical, date or language restriction in the selected databases. The references cited by each included article will be reviewed for potentially eligible studies. Data extraction to be completed in a predefined data extraction sheet. Terms used for the literature search were: Aedes aegypti” OR “Aedes albopictus” OR “Aedes” AND “Dengue” OR “Chikungunya” OR “Zika” combined with “Urbanization” and “Human Population Density”. Searches were conducted with a combination of all keywords including at least the term “Urbanization” or “Human Population Density”.

Condition or domain being studied

The aim of this study is to evaluate the impact of urbanisation on the emergence and re-emergence of Aedes-borne infectious diseases, and the degree to which urbanisation processes create suitable habitats for Aedes mosquitoes.

Intervention(s), exposure(s)

Inclusion criteria: i) Studies examining how/if urbanization affects the density, larval development rate, breeding sites and adult survival time of Aedes albopictus and Aedes aegypti, ii) Studies examining how/if urbanization affects the establishment, distribution and vector density of Aedes albopictus and Aedes aegypti, iii) Studies examining how/if human population density, favours the

spread of dengue, zika and chikungunya incidence cases and epidemics, iv) Studies examining how/if geo-spatial patterns of urbanization or human population density affect the seroprevalence and incidence of Aedes borne diseases, v) Studies examining how/if the urbanization trends create a dynamic and favor the incidence of dengue, chikungunya or zika.

Exclusion criteria: i) Studies not including urbanization or population density as a potential predictor variable or an explanatory variable for the incidence of dengue, chikungunya or zika or Aedes population spread, ii) Studies focusing on Yellow Fever and Urbanization or Population Density, iii) Studies focusing on mosquito species other than Aedes albopictus or Aedes Aegypti, iv) Studies focusing on Knowledge Attitude Practices or clinical and laboratory characteristics with minor reference to Urbanization or Population Density, v) Studies conducted in urbanization settings consisting of a human population density less than 1000 inhabitants per square kilometer vi) Opinion papers or Descriptive studies not providing numerical values or correlation indexes.

Definitions of "urban" may vary within the existing literature. From our search results, we will identify different components of urbanisation and will categorise Aedes ecology and disease transmission by the definitions. This will allow us to identify those components of urbanisation that lead to those outcomes.

Types of study to be included

Entomological; Epidemiological; Ecological studies and Models; Studies of populations in URBAN areas; Studies conducted in urbanised settings consisting of a human population density MORE than 1000 inhabitants per square kilometre

Primary outcome(s)

The effect of urbanization will be studied mainly through the population density criterion and confirmed through the overall urbanization context of the study area.

Association of Aedes ecology and transmission dynamics for dengue chikingunya and zika will be studied according to the following non-exhaustive variables:
population density;
urban growth;
urban vs rural gradient;
geospatial characteristics;

Data extraction

Two reviewers will independently screen titles and abstracts to identify all sources appear to meet the inclusion criteria or where there is any uncertainty. A third reviewer will be used in the case of any disagreements. Reviewers will obtain the full report of the included sources to confirm that it meets the inclusion criteria.

Risk of bias (quality) assessment

The variety of methodological approaches of studies included in the analysis will be assessed by

the use of a mixed-methods tool for appraising different qualitative and quantitative aspects of the included studies. The mixed methods approach and tool will be considered for its potential to assess the methodological quality of quantitative, qualitative, and mixed-methods studies.

Strategy for data synthesis

The planned data synthesis will be narrative (descriptive), and based on the effects of urbanization on Aedes ecology and Aedes borne disease transmission dynamics.
We will provide summaries of each category by study type, methods, human population density, urban characteristics of included studies, measured outcomes, findings and conclusions. Findings in the two categories (Aedes ecology and Aedes borne disease transmission dynamics) will be allocated to further categories for comparable analysis.

Analysis of subgroups or subsets

We also plan to assess how common different characteristics of URBAN found in the literature are associated with Aedes ecology and disease dynamics

Type and method of review

Systematic review

Language

English

Country

Greece, Germany, UK

Keywords

Aedes, urbanisation, systematic review

Current review status

Ongoing
